# The horror of today and the terror of tomorrow: The role of future existential risks and present‐day political risks in climate activism

**DOI:** 10.1111/bjso.12821

**Published:** 2024-11-04

**Authors:** Mete Sefa Uysal, Nuria Martinez, Sara Vestergren

**Affiliations:** ^1^ University of Exeter Exeter UK; ^2^ Canterbury Christ Church University Canterbury UK; ^3^ Keele University Newcastle‐under‐Lyme UK

**Keywords:** climate crisis, collective action, future, politicized identity, risk perception

## Abstract

In response to the urgent global climate crisis, climate activism has risen as a potent force. Decision‐making regarding climate collective action includes individuals' perceptions of the anticipated future existential risks of the climate crisis (*risk of inaction*) and present‐day political risks of climate activism (*risk of action*). Our research, spanning four studies (two correlational surveys and two pre‐registered experiments), focused on climate activism in Germany (*N* = 1027). We consistently showed that heightened politicized activist identification was associated with both confrontational and non‐confrontational climate collective action across four studies. Furthermore, the anticipated existential climate risk was associated with non‐confrontational climate action and present‐day political risk with confrontational action. Politicized climate identity remained a robust predictor across different action tactics, while the content and temporality of risk (future existential vs. present‐day political) in one's environment determined the transition between engagement in confrontational and non‐confrontational climate action dynamically. Nevertheless, we did not find causal links between risk perceptions and collective action. We discuss our findings in line with ESIM (Elaborated Social Identity Model), and potential explanations for the lack of causal relationship and future directions for alternative methodologies and comprehensive conceptualization of risk perceptions are suggested.

## BACKGROUND

Threatened with the prospect of a bleak future, we imagine alternative ones that we wish to collectively bring about. Climate activism is a case in point. It signifies a future‐oriented movement directed at addressing a global challenge that will inevitably affect everyone. Extending beyond weather events and environmental disasters, the climate crisis impacts industries, job markets, and the cost of living (Chomsky & Pollin, 2020; Macintosh, 2022). Additionally, it contributes to worsened air quality, the spread of diseases (Williams et al., 2021), decreased mental health (Hrabok et al., 2020), changes in migration patterns (Ferris, 2020), and inter‐generational conflicts (Han & Ahn, 2020). Consequently, climate activism plays a crucial role in bringing about a more sustainable future; it represents future‐oriented action to strive away from multifaceted risks posed by a negative yet globally shared future. As previous research has highlighted, concern for future generations (Neas et al., 2022), apocalyptic visions of the future (Cassegård & Thörn, 2018) and perceptions of existential and irreversible climate threat (Uysal et al., 2022; Uysal, Vestergren, et al., 2024) are all key drivers of climate activism.

This paper adds to this line of research by bringing attention to present‐day risks associated with climate change (in)action. In doing so, this research seeks to examine the impact of conflicting risk perceptions, such as the anticipated existential future risk of climate crisis (*risk of inaction*) and present‐day political risks of climate activism (*risk of action*), in addition to group consciousness (politicized social identity and efficacy beliefs), on the climate collective action intention in Germany. The paper spans four studies utilizing correlational and experimental methods. The goal is to gain insights into how individuals make decisions when faced with motivating and de‐motivating risk perceptions within the context of the climate crisis. Through this research, we aim to provide comprehensible insights into the factors related to risk and identity that either drive or impede individuals' involvement in climate‐related collective action.

### Political and existential risk perceptions

The way we interpret risks in our surroundings can produce various coping responses, including taking action to address or resist the risk or opting to avoid the issue altogether (Pidgeon, 2012). The term “risk perception” pertains to the way individuals or groups comprehend and assess potential risks or threats in their environment (Slovic, 2016). This involves subjectively evaluating the likelihood and severity of various hazards, uncertainties, or negative outcomes. Risk perceptions are shaped by various factors, such as personal experiences, group dynamics, cultural beliefs, information sources, and cognitive biases (Poortinga et al., 2019; van der Linden, 2014, 2015). Understanding people's perceptions of risks is crucial for understanding and influencing decision‐making, policy formulation, and effective communication. It aids in evaluating and addressing concerns and informs the development of strategies for risk mitigation and management (Poortinga & Pidgeon, 2003; Whitmarsh, 2009).

Perceptions of potential risks in social and political life, as well as the influence of these risk perceptions on motivations for collective action, remain relatively understudied in contemporary social psychology research (see Klandermans, 1997; Klandermans & Oegema, 1987; Olson, 1965 for historical exceptions). Yet, the way in which people perceive the police and law as representatives of state power can significantly impact perceptions of risk to life, body integrity, future, and freedom during conflictual collective actions and crowd events such as climate protests (Ayanian & Tausch, 2016; Uysal et al., 2022). In certain cases, these political risk perceptions may override other psychological variables that typically encourage participation in collective action, such as perceived efficacy, anger, and perceived injustice. For example, in authoritarian contexts like Russia, Hong Kong, and Turkey, individuals' perception of the likelihood of political and activism‐related risks is associated with heightened fear (Ayanian et al., 2021). This fear can dissuade individuals from participating in actions perceived as risky or dangerous, inhibiting collective action more broadly (Adra et al., 2020). On a broader scale, when authoritarian states employ repressive political measures, such as the use of force against social movements and collective actions, it can hinder the translation of psychological motivations, such as self‐efficacy and environmental concern, into climate activism across multiple countries (Uysal, Vestergren, et al., 2024). Consequently, perceived political risks stemming from states' repressive measures and police violence may suppress people's willingness to engage in collective action, even when they hold strong beliefs and motivations related to the movement or action cause (Uysal et al., 2022).

In addition to perceiving political risks linked to taking action (such as risk perception related to confrontations with the police or state), activists may also recognize existential risks tied to inaction (e.g., climate concern and anticipation of climate catastrophes) that may be in conflict with the aforementioned political risks. Engaging in environmental collective action, for instance, might serve as a coping strategy against negative emotions arising from environmental concerns (Vestergren et al., 2018, 2022). Intentions to partake in action against the climate crisis can be an outcome of heightened fear regarding the future negative consequences of the climate crisis (van Zomeren et al., 2010). As an example, recent research has demonstrated that feeling concerned about environmental issues correlates with involvement in environmental collective action in the last 5 years across various countries (Uysal, Vestergren, et al., 2024).

Thus, the interplay between risk perception and climate activism suggests a complex landscape wherein individuals grapple with conflicting perceptions of risk related to both action and inaction. This conflict serves as a focal point for understanding the motivations and hesitations of activists addressing climate change. The fundamental risk perception of conflict revolves around the uncertainty of whether the perceived existential risks of inaction will indeed drive action when weighed against the political risks associated with taking action. Activists, confronting risks of repression, police brutality, and sanctions, alongside the risks tied to climate change and policy inaction, must navigate this complex decision‐making process. This conflict not only shapes individual coping responses but also influences the trajectory and sustainability of collective actions.

### Social identity at the core of risk perceptions

Understanding the complex relationship between political and existential risk perceptions and climate collective actions necessitates considering the role of social identity. Social identity, a crucial component of one's self‐concept derived from perceived membership in relevant social groups (Tajfel & Turner, 1979; Turner et al., 1987), plays a pivotal role in collective action. Collective action is often rooted in shared norms and values arising from identification with a social group (Drury & Reicher, 2000). How we define ourselves through collective identities influences when and how we engage in specific collective actions (Uysal, Saavedra, & Drury, 2024). If an issue aligns with the norms and values of a social identity, members of that group are more mobilized to act. Conversely, if the issue is unrelated or antagonistic to the social identity, mobilizing group members becomes more challenging. Politicized social or collective identities refer to social identities defined in terms of a political cause or struggle by members of that group (Simon & Klandermans, 2001). Activists often operate within a normative framework of their politicized social identities. In essence, when individuals feel and think as members or representatives of a particular politicized group, they have a framework of norms and beliefs regarding what should be done against a crisis or problem and how it should be done based on the salient social identity.

Framing collective actions as intergroup encounters, The Elaborated Social Identity Model of Crowd Behaviour (ESIM; Drury & Reicher, 2000; Stott & Reicher, 1998) suggests that shared (politicized) identity defines appropriate and legitimate conduct within collective actions. According to this model, police intervention (as a present‐day political risk) perceived as illegitimate by ingroup members can reshape in‐group norms, motivating defensive responses, and potentially leading to a more radical self‐concept and identity (Vestergren et al., 2019). Conflictual interactions can establish new in‐group norms that allow for novel strategies that seem radical by the majority of that society or police and policymakers in particular. Activists with prior conflictual police interactions may anticipate more political risk and anticipation of police violence in the protest; hence, they could demonstrate a stronger intention to engage in confrontational collective action, which involves actions like occupying, blocking traffic, and eco‐vandalism. This suggests that, in the face of risks, politicized social identity does not only influence collective action participation but also the collective action tactic that will be adopted.

Furthermore, in addition to the direct influence of politicized social identity on collective action (and adopted collective action tactics), politicized social identity could mobilize people by amplifying the perception of injustice, anger, perceived collective efficacy and empowerment (Drury & Reicher, 2005; van Zomeren et al., 2008) or buffering negative emotions like fear (Adra et al., 2020) and risk perceptions (Ayanian et al., 2021; Ayanian & Tausch, 2016). The buffering effect of a politicized social identity can manifest in two ways. First, it can diminish the subjective importance of a perceived *risk of action* (e.g., Ayanian et al., 2021; Ayanian & Tausch, 2016). For instance, Ayanian and Tausch (2016) demonstrated that identification with a politicized identity among activists during the Arab Spring in Egypt predicted greater willingness to participate in future protests, as the subjective importance of risks stemming from government sanctions was reduced. Second, it can elevate the significance of the *risk of inaction* (e.g., Uysal et al., 2022) that motivates participation in collective action. In the context of climate activism, this *risk of inaction* is referred to as the future existential risk of the climate crisis, that is, the expectations of environmental catastrophes happening in the future and risk perceptions arising due to these expectations. Altogether, politicized social identity serves as a driving force, encouraging participation while acting as a buffer against adverse political risks. Furthermore, it amplifies the existential risks associated with the climate crisis, fostering a heightened sense of urgency and commitment to taking action.

Efficacy beliefs stemming from social identity (Thomas et al., 2012) could serve as another critical component of collective belonging, creating a buffer effect for adverse political risks and an amplifier effect for existential climate risks. Collective or group efficacy, defined as the belief in achieving shared goals through collective efforts, is a well‐documented predictor of collective action (Agostini & van Zomeren, 2021; Mummendey et al., 1999). Recent research has highlighted the importance of participative efficacy beliefs in collective action, which emphasize the role of individual contributions in achieving group goals (Masson & Fritsche, 2021; Mazzoni & Cicognani, 2015; van Zomeren et al., 2013). Whether believing in the collective ability of a group to achieve common goals or in one's own individual contribution to these collective efforts, people assess these efficacy beliefs in the face of various risk perceptions. Believing in one's individual or group's capacity to bring about change by overcoming challenges and obstacles helps to counteract the adverse impact of risks of actions. Therefore, we will measure efficacy beliefs as control variables while examining the role risk perceptions on collective actions.

## CONTEXT AND CURRENT RESEARCH

In recent years, climate activism in Germany has been largely influenced by the global climate movement, spearheaded by figures like Greta Thunberg. Youth‐led movements such as Friday for Future have gained widespread attention and mobilized thousands of students and young people to demand stronger action on climate change from policymakers. Current public and political discussions in German climate activism often revolve around the urgency of climate action and the normativity and efficacy of different collective action tactics such as civil disobedience, disruptive and confrontational protests by groups like *Ende Gelände* and *Letzte Generation*, as opposed to so‐called normative and peaceful tactics by groups like *Friday for Future*, and finally how the police should react to climate collective actions (Connally, 2023; Niranjan, 2023; Weise, 2023).

Recent years have witnessed environmental activists employing unconventional tactics in Germany, including targeting famous artworks and disrupting traffic flow to draw attention to the climate emergency. In response to the increased disruptive protests such as eco‐vandalism and cutting off traffic, German authorities have called for more stringent measures and supported use‐of‐force by police (Connally, 2023). While most of the climate protests in Germany are peaceful and conducted without police intervention, there have been an increasing number of incidents where police have used pepper spray, batons and arrested activists heavy‐handedly using force.

These discussions around increased political risks and the urgency of climate actions provide us with an opportunity to examine the role of conflicted political and existential risk perceptions on climate collective action engagement. Across two correlational and two pre‐registered experimental studies, we investigate whether higher present‐day political risk perception (*risk of action*) is associated with a lower willingness to engage in climate action in Germany, while higher future existential risk perception (*risk of inaction*) is linked to stronger intentions for climate collective action. We also aim to explore the interaction (or conflict) between these different risk perceptions. Furthermore, we examine the role of politicized climate activist identity and efficacy beliefs in climate collective action. Lastly, we assess the role of these variables on participation in different collective action tactics (confrontational vs. non‐confrontational) (Study 2–4).

## STUDY 1

### Method

#### Participants

All data and codes are openly accessible in OSF: https://osf.io/hv8ks/. We collected data online in January 2023 in Germany. Data were collected from participants who live in Germany, fluently speak German, and were over 18 years of age. A priori power analyses showed that we needed a minimum of 253 participants to attain a power of .95 for small to moderate effect size (*f* ^2^ = .08) with an *α* error probability level of .05 to perform linear regression analysis using five predictors (i.e., activist identity, collective and participative efficacy, and risk perceptions). Using convenience and snowball sampling through social media, we distributed the link to the survey on mail groups for psychology students at Friedrich Schiller University Jena and the personal network of the researchers and research assistants. The survey was completed by 325 participants. Participants' average age was 24.57 (SD = 10. 56). Sixty‐eight participants were self‐identified male, 245 females, seven were non‐binary, and seven did not want to answer (Table 1).

**TABLE 1:** Sample demographics across studies.

	Study 1 (*n* = 325)	Study 2 (*n* = 298)	Study 3 (*n* = 182)	Study 4 (*n* = 222)
Age	24.6 (10.6)	24.3 (9.1)	24.4 (8.5)	25.7 (11.6)
Gender				
Female	245	216	131	160
Male	68	76	46	49
Non‐binary	7	5	5	10
No answer	7	2	–	3
Region				
Former East German States	255 (78.5%)	232 (77.9%)	174 (95.6%)	184 (82.9%)
Former West German States	70 (21.5%)	66 (22.1%)	8 (4.4%)	38 (17.1%)
Political orientation (from 1 = left to 10 = right)	2.8 (1.7)	2.5 (1.6)	3.8 (1.7)	2.7 (1.7)

*Note*: Age and political orientation show mean scores, standard deviations are in brackets. Gender and region demonstrate frequencies for each category.

#### Measures

We used 5‐point response scales (from 1 = strongly disagree to 5 = strongly agree) unless otherwise stated. For all scales, the order of presentation of the items was randomized.

##### Climate activist identity

We measured climate activist identification with five items (Leach et al., 2008; Postmes et al., 2013): “I identify myself as a climate activist,” “I feel a bond with climate activists,” “I am glad to be a climate activist,” “I often think about the fact that I am a climate activist” and “I have a lot in common with other climate activists” (*α* = .88).

##### Group efficacy

Group efficacy is measured with three items (van Zomeren et al., 2010): “We can mitigate climate crisis together,” “We can fight against climate crisis through joint actions” and “We can achieve our common goals in fighting against climate crisis” (*α* = .77).

#### Participative efficacy

We measured participative efficacy with three items (van Zomeren et al., 2013): “I as an individual can provide important contribution so that we can mitigate climate crisis together,” “I as an individual can provide a significant contribution so that we can fight against climate crisis through joint actions” and “I as an individual can contribute meaningfully so that we can achieve our common goals in fighting against climate crisis” (*α* = .83).

##### Future existential risk of climate crisis

Risk anticipation related to the climate crisis is measured with two items (Uysal et al., 2022): “How do you evaluate the likelihood of the global climate crisis causing the extinction of species?” and “How do you evaluate the likelihood that the global climate crisis will have a negative impact on people's lives in the coming years?” (from 1 = *very unlikely* to 5 = *very likely*; *r* = .66).

##### Present‐day political risk of climate activism

We measured perceived likelihood of political risk in the context of climate protests with two items (Ayanian & Tausch, 2016; Uysal et al., 2022): “How do you evaluate the likelihood of being arrested or detained if you participate in action against the climate crisis?” and “How do you evaluate the likelihood of facing police brutality/violence if you participate in action against the climate crisis?” (from 1 = *very unlikely* to 5 = *very likely*; *r* = .69).

#### Climate collective action

We measured willingness to participate in collective action to mitigate climate crisis with six items: I would be willing to “become a member of an organization that works for ecological justice,” “send a protest letter to authorities to warn them of their actions that could accelerate climate crisis,” “boycott products (e.g., meat, dairy, cosmetics, clothing, etc.) that accelerate the climate crisis,” “participate in a demonstration for ecological justice,” “participate in protests that need commitment to defend ecological justice (e.g., *Hambacher Forst*, *Lützerath*)” and “participate in the blocking of factories, companies etc. that stand in the way of ecological justice.” Principal component analysis showed a single‐factor structure (*α* = .85).

### Results and discussion

The descriptive statistics and zero‐order correlations between variables are depicted in Table 2. Inspection of the means through a one‐sample *t*‐test shows that, on average, participants reported higher levels of future existential risk of climate crisis (*M* = 4.50, *t* = 37.08, *p* < .001), participative efficacy (*M* = 3.26, *t* = 4.68, *p* < .001), group efficacy (*M* = 3.63, *t* = 13.11, *p* < .001), and willingness to participate in climate collective action (*M* = 3.13, *t* = 2.40, *p* = .017) compared with the scales' mid‐points. However, they reported lower levels of present‐day political risk of climate activism (*M* = 2.53, *t* = −8.49, *p* < .001) and activist identity (*M* = 2.42, *t* = −11.34, *p* .001).

**TABLE 2:** Means, standard deviations, and correlations of all measures, Study 1.

Variables	*M* (SD)	1	2	3	4	5	6
1. Collective action	3.13 (.98)	–	.72***	.29***	.22***	.51***	.72***
2. Activist identity	2.42 (.92)		–	.28***	.30***	39***	29***
3. Group efficacy	3.63 (.86)			–	.55***	.28***	.09
4. Participative efficacy	3.26 (1.00)				–	.19**	.08
5. Future existential risk of climate crisis	4.50 (.73)					–	.22***
6. Present‐day political risk of climate activism	2.53 (.99)						–

*** *p* < .001.

** *p* < .01.

Then, a linear regression analysis was carried out in SPSS to examine the degree to which activist identification, group and participative efficacy, and future existential risk of the climate crisis and present‐day political risk of climate activism would be linked to willingness to participate in climate collective action (Table 3). Stronger activist identity (*b* = .64, SE = .04, *p* < .001) and future existential risk of climate crisis (*b* = .34, SE = .04, *p* < .001) predicted a higher willingness to participate in climate collective action. Efficacy beliefs and present‐day political risk of climate activism did not predict climate collective action.

**TABLE 3:** Model summary of regression analysis, Study 1.

	*b*	SE	*β*	*t*	*p*
Activist identity	.64	.04	.60	14.49	<.001
Group efficacy	.09	.05	.08	1.70	.091
Participative efficacy	−.05	.04	−.05	−1.23	.219
Future existential risk of climate crisis	.34	.05	.25	6.33	<.001
Present‐day political risk of climate activism	.04	.04	.05	1.19	.236
*F*	90.137				
*R* ^2^	.59				

Furthermore, for explanatory purposes, we conducted mediation analyses to test the mediating roles of the present‐day political risk of climate activism and the future existential risk of the climate crisis on the relationship between politicized activist identity and collective action intention using the bootstrap resampling method in R lavaan (Rosseel, 2012). Although we acknowledge that the mediation analysis in correlational data cannot imply any kind of causal relationship, we aim to explore whether different risk perceptions mediate the relationship between identification and collective action differently. Activist identity predicted collective action through the future existential risk of the climate crisis (*b* = .16, SE = .03, 95% CI [0.10, 0.22], indirect/total effect = 17.20%). However, the indirect effect of activist identity on collective action intention through present‐day political risk was not significant, *b* = .02, SE = .02, 95% CI [−0.03, 0.07]. Overall, future existential risk, but not present‐day political risk, mediated the positive relationship between politicized activist identity and collective action intention.

Our results imply that individuals who internalize politicized climate activist identity more, or in other words, those who feel a strong connection to the goals and values of the climate movement, are more likely to actively participate in efforts to address the climate crisis. Moreover, Study 1 showed the role of the existential threat posed by climate crisis in stronger intention to participate in climate collective action. However, one important limitation of this study is that collective action is conceptualized and measured in a limited way, overlooking different collective action tactics. To address this, in the second study, we aim to replicate and extend Study 1, focusing on both confrontational and non‐confrontational collective action.

## STUDY 2

Collective action involves group members acting as representatives to enhance group conditions and takes on various forms, ranging from non‐confrontational actions to confrontational and disruptive actions (Uysal, Saavedra, & Drury, 2024; Wright et al., 1990). Confrontational collective actions—variously defined as non‐normative, radical, aggressive, or violent—often involve civil disobedience, disruptive activism, conflict escalation, and unauthorized demonstrations (Uysal, Saavedra, & Drury, 2024). It entails a range of actions in which activists enact their group identity, identity‐motivated group goals, and grievances in the presence of outgroup members (e.g., police) or with the anticipation of outgroup intervention, regardless of whether physical conflict occurs. These actions may include riots, high‐risk protests, occupations, property damage, traffic blockades, unauthorized marches, or counter‐protests, often characterized by the potential for escalating intergroup conflict. Activist groups, however, typically do not view confrontational and non‐confrontational actions as opposing strategies. Instead, they consider them part of a broad political repertoire, employing both approaches simultaneously or at different times (Penic et al., 2024; Zúñiga et al., 2023). Transition between action tactics depends on anticipated political and existential risks at that time, particularly within the context of climate activism. In the second study, we broadened the measurement of our outcome variable: collective action. The collective action measure of the first study mostly focused on non‐confrontational collective action and principal component analysis showed that the items form a single factor structure. In the second study, we replicate and extend the findings of the first study by testing to what extent identity, efficacy beliefs, and risk perceptions predict both confrontational (e.g., blocking factories, damaging properties) and non‐confrontational (e.g., becoming a member of an organization, participating demonstration) climate collective action.

### Method

#### Participants

We collected data online in January and February 2023 in Germany. We followed the same inclusion and exclusion criteria and a similar recruitment strategy as in Study 1. A total of 298 participants completed the survey. Participants' average age was 24.33 (SD = 9.07). Seventy‐five participants were self‐identified male, 216 females, five non‐binary, and two did not answer.

#### Measures

We used 5‐point response scales (from 1 = strongly disagree to 5 = strongly agree) unless otherwise stated. We measured activist identity (*α* = .88), group efficacy (*α* = .70), participative efficacy (*α* = .83), future existential risk of climate risk (*r* = .59), and present‐day political risk of climate activism (*r* = .64) as in the first study.

##### Confrontational climate collective action

We measured willingness to participate in confrontational collective action to mitigate climate crisis with four items: I would be willing to participate “in occupying action for ecological justice,” “in protests that need commitment to defend ecological justice (e.g., Hambacher, Forst, Lützerath),” “in blocking of factories and companies that stand in way of ecological justice” and “damage property or goods that stand in the way of ecological justice” (*α* = .89).

##### Non‐confrontational climate collective action

Willingness to participate in non‐confrontational collective action was assessed with four items: I would be willing to “become a member of an organization that works for ecological justice,” “participate in a demonstration for ecological justice,” “join solidarity farming or energy cooperatives” and “provide educational events concerning ecological justice” (*α* = .80).

Before deciding on items for confrontational and non‐confrontational collective actions, a group of students engaged in climate activism in Germany was asked for their feedback and suggestions on common confrontational and non‐confrontational actions in the climate movement. During the discussion, solidarity actions with ecological communes, sit‐in and occupy actions, damaging properties such as attacking the company buildings and throwing paints in certain statues and buildings, and long‐term resistance against deforestation that requires conflict with police and demolition vehicles were considered and students share their insights on such actions. Based on their input, eight items were developed. We conducted a principal factor analysis using eight items of confrontational and non‐confrontational climate collective action to test whether items form a two‐factor structure. As we reported above, collective action items are loaded on two factors named confrontational and non‐confrontational collective action (KMO = .89; Barlett's test of sphericity: *x* ^2^ (28) = 1284.68, *p* < .001; total variance explained 69.64%). Items loadings ranged from .69 to .85 for confrontational collective action and .72 to .76 for non‐confrontational collective action.

### Results and discussion

The descriptive statistics and zero‐order correlations between variables are depicted in Table 4. Inspection of the means through one‐sample *t*‐tests shows that, on average, participants reported lower levels of activist identity (*M* = 2.55, *t* = −8.23, *p* < .001), present‐day political risk of climate activism (*M* = 2.55, *t* = −8.04, *p* < .001), and willingness to participate in confrontational collective action (*M* = 2.33, *t* = −10.70, *p* < .001), whereas higher levels of group efficacy (*M* = 3.58, *t* = 12.60, *p* < .001), future existential risk of climate crisis (*M* = 4.29, *t* = 27.65, *p* < .001), and non‐confrontational collective action (*M* = 3.28, *t* = 4.75, *p* < .001), compared with the scales' mid‐points.

**TABLE 4:** Means, standard deviations, and correlations of all measures, Study 2.

Variables	*M* (SD)	1	2	3	4	5	6	7
1. Confrontational collective action	2.33 (1.07)	–	.65***	.64***	.27***	.10	.24***	.33***
2. Non‐confrontational collective action	3.28 (1.00)		–	.60***	.38***	.25***	.41***	.28***
3. Activist identity	2.55 (.95)			–	.37***	.20**	.21***	.27***
4. Group efficacy	3.58 (.79)				–	.48***	.30***	.20**
5. Participative efficacy	3.08 (.96)					–	.14*	.05
6. Future existential risk of climate crisis	4.29 (.80)						–	.24***
7. Present‐day political risk of climate activism	2.55 (.96)							–

*** *p* < .001.

** *p* < .01.

* *p* < .05.

To test to what extent politicized climate activist identity, efficacy beliefs, and risk perceptions predict confrontational and non‐confrontational collective action, we performed two linear regression analyses (Table 5). Willingness to participate in confrontational collective action is predicted by a stronger climate activist identity (*b* = .66, SE = .06, *p* < .001) and present‐day political risk of climate activism (*b* = .17, SE = .05, *p* = .002). Willingness to participate in non‐confrontational collective action is predicted by stronger climate activist identity (*b* = .65, SE = .06, *p* < .001) and future existential risk of climate crisis (*b* = .33, SE = .06, *p* < .001).

**TABLE 5:** Model summary of regression analyses, Study 2.

	Confrontational					Non‐confrontational				
	*b*	SE	*β*	*t*	*p*	*b*	SE	*β*	*t*	*p*
Activist identity	.65	.06	.58	12.03	<.001	.51	.05	.48	10.23	<.001
Group efficacy	.02	.07	.02	.33	.742	.09	.07	.07	1.29	.198
Participative efficacy	−.05	.06	−.04	−.84	.401	.09	.05	.08	1.71	.089
Future existential risk of climate crisis	.12	.06	.09	1.84	.068	.33	.06	.26	5.68	<.001
Present‐day political risk of climate activism	.17	.05	.15	3.16	.002	.08	.05	.08	1.65	.102
*F*	45.940					50.854				
*R* ^2^	.44					.47				

Next, we conducted mediation analyses as in Study 1 for explanatory purposes. We aim to explore whether different risk perceptions mediate the relationship between identification and different collective action tactics. While both present‐day political risk of climate activism (*b* = .07, SE = .03, 95% CI [0.01, 0.12], indirect/total effect = 8.38%) and the future existential risk of climate crisis (*b* = .04, SE = .03, 95% CI [0.00, 0.19], indirect/total effect = 4.76%) mediated the relationship between activist identity and confrontational collective action, activist identity predicted confrontational collective action through present‐day political risk more strongly than future existential risk. Furthermore, both future existential risk of climate crisis (*b* = .09, SE = .04, 95% CI [0.00, 0.19], indirect/total effect = 12.07%) and present‐day political risk of climate activism (*b* = .07, SE = .03, 95% CI [0.01, 0.13], indirect/total effect = 8.65%) mediated the relationship between activist identity and non‐confrontational collective action intention, while activist identity predicted non‐confrontational collective action through future existential risk of climate crisis more strongly than present‐day political risk of climate activism.

Consistent with the results of Study 1, our findings indicate that a stronger climate activist identity plays a significant role in motivating individuals to engage in both confrontational and non‐confrontational forms of collective action. This suggests that individuals who identify strongly with the goals and values of the climate movement are more likely to participate actively in addressing climate change, regardless of the specific tactics involved.

Study 2 also highlights the distinct role of risk perceptions in different domains and temporality (future existential vs. present‐day political) in shaping willingness to engage in different forms of collective action. While the future existential risk of climate crisis emerged as a significant predictor of willingness to participate in non‐confrontational collective action, the present‐day political risk of climate crisis emerged as a key predictor of willingness to engage in confrontational tactics. According to ESIM (Drury & Reicher, 2000; Stott & Reicher, 1998), illegitimate and indiscriminate actions of police towards both radicalized and non‐radicalized crowds could be the core of the normative change. The perceived unjust and indiscriminate actions of the police can influence the norms of activist groups, developing radicalization in their protest tactics. The present‐day political risk anticipated by activists, such as sanctions, might include the perception of indiscriminate actions of the police, as they will face the same use of force from police regardless of their protest tactics. When activists anticipate political risks regardless of their tactics, they might as well choose confrontational tactics, as these are perceived to be more effective in pressuring governments for urgent changes compared to non‐confrontational actions, given the same high risk but potentially higher gains of confrontational collective action (Li et al., 2024; Zúñiga et al., 2023). Overall, the findings suggest that individuals may weigh different types of risks differently when considering their involvement in different collective action tactics.

## STUDY 3

In the third study, we conducted a pre‐registered experiment to infer a causal link between perceived risk and climate collective action (https://osf.io/bm76p/?view_only=1f485a78253944a1af7e2dffba8720b3). We examined how different risk perceptions influence willingness to participate in future climate collective action. We explored the role of both future existential risks of climate crisis (e.g., anticipation of climate catastrophes) and present‐day political risks of climate activism (e.g., police brutality and repression). By manipulating the level of future existential risks of the climate crisis and present‐day political risks of climate activism, we tested their unique effect on both willingness to engage in confrontational and non‐confrontational climate collective action, in addition to its interaction effect. To do so, we conducted a 2 × 2 between‐subject factorial experiment. Furthermore, we tested to what extent activist identity and efficacy beliefs are associated with confrontational and non‐confrontational climate collective action, as in Study 2.

### Method

We collected data online in May and June 2023 in Germany. We used the same data collection strategy and inclusion/exclusion criteria in the first two studies. Using the smallest correlations between risk and climate collective action variables in the first two studies, power analysis was conducted for the minimum number of observations required to examine the research hypotheses. Analyses showed that at an α value of .05, a power of .80, and a two‐way H0 hypothesis, a minimum of 128 participants should be reached. First, 492 participants took part in the study. After we removed the participants for who we did not have full data on the measures of interest and did not spend enough time on the manipulation task, the final sample was 182. Participants' average age was 24.40 (SD = 8.45). Forty‐six participants were self‐identified male, 131 female, and five non‐binary. From 1 = left to 10 = right, participants' political orientation on average was 3.79 (SD = 1.73).

#### Measures

We used 5‐point response scales (from 1 = strongly disagree to 5 = strongly agree) unless otherwise stated. We measured activist identity (*α* = .92), group efficacy (*α* = .84), participative efficacy (*α* = .89), confrontational (*α* = .92) and non‐confrontational climate collective action (*α* = .85) as in Studies 1 and 2. Confrontational and non‐confrontational climate collective action items were from two confirmatory components as in Study 2. We also measured the future existential risk of climate crisis (*r* = .65) and present‐day political risk of climate activism (*r* = .73) as in previous studies to test the functionality of manipulation.

#### Procedure and manipulation task

Participants provided their informed consent and demographic information. Then, they completed activist identity and efficacy scales in a randomized order. Subsequently, participants were randomly assigned to one of the four experimental conditions. Following their response to the manipulation check questions, they proceeded to complete confrontational and non‐confrontational climate collective action scales in a randomized order.

Although confidence in the media has been declining over the past years, Germany maintains one of the highest trust in media across Europe, with between 43% to 53% of Germans expressing trust in news (Hölig, 2023). Therefore, we used fictional newspaper articles to manipulate risk perceptions. A fictional newspaper article was created in which the future existential risk of climate crisis (low vs. high) and present‐day political risk of climate activism (low vs. high) could be manipulated. After a very brief introduction to the climate crisis, a piece from an interview with a fictitious esteemed researcher summarizes their new findings on the climate crisis and its effects on Germany. The high future existential risk of climate crisis condition describes how the destructive consequences are bigger than expected:

The ice glaciers in the European Alps are experiencing a huge toll, and rain instead of snow is falling there. The number of extreme weather events has more than tripled in Germany over the past 50 years. Days of forest fires and floods have already destroyed the habitats of thousands of people in Europe. These events massively accelerate species extinction.

In contrast, the low future existential risk of climate crisis condition summarizes how new research findings are more optimistic than previous predictions; hence, the effects of the climate crisis won't be as destructive:

The impact of extreme weather events is going to be far smaller than expected. We have already reached 70% of the maximum possible global warming due to man‐made climate change. This means that temperatures are not going to rise higher than 1.4°C in the future if we all keep doing what we're doing. This pattern may slow down species extinction.

In the present‐day political risk of climate activism manipulation, the high political risk condition describes a recent police attack on a peaceful climate demonstration in Berlin and a recent report that shows violence and sanctions against climate activists are rising in Europe:

Berlin hosted the biggest climate strike of recent years. While police used pepper spray and tear gas against thousands of climate activists who were peacefully demonstrating for urgent policy changes for clean energies, several protesters were hospitalized. This incident supported the recent report that showed police forces in Germany, England, and France increasingly show violence against climate activists, and the courts of these countries sentence activists more frequently. As a result of this increased police violence, the safety of the activists has come into question.

On the other hand, the low political risk condition describes the opposite:

A record number of activists participated in this peaceful demonstration. This strike showed once again that an increasing number of activists are engaging in the climate movement since the law amendment restricted police attacks in climate strikes in Europe. People can now freely and safely participate in the actions and show efforts against the climate crisis.

### Results and discussion

First, to examine whether the manipulation worked, future existential risk and present‐day political risk items used in the first two studies are employed as manipulation checks. The results of the Mann–Whitney *U*‐test analysis show that participants in the present‐day political risk of climate activism condition (*M* = 2.63, SD = 1.02) evaluated the present‐day political risk higher than participants in the low protest risk condition (*M* = 2.39, SD = 0.95), *U* = 3583, *p* < 0.001, indicating that the manipulation worked for present‐day political risk. However, our manipulation for future existential risk failed to create the intended effect on participants. Participants in the high future existential risk of climate crisis condition (*M* = 3.93, SD = 0.96) evaluated the future existential risk of climate crisis slightly higher than participants in the low existential risk condition (*M* = 3.87, SD = 0.89), but this difference did not reach statistical significance, *U* = 3859, *p* = .432.

As the future existential risk manipulation failed, we reported our analysis only for present‐day political risk manipulation (for details on other analyses, see Appendix S1). To examine the influence of the present‐day political risk of climate activism on confrontational and non‐confrontational collective action intention, we conducted ANCOVA with identification, collective efficacy, and participative efficacy as control variables, and Mann–Whitney *U*‐tests. While politicized activist identity was associated with stronger confrontational collective action (*F*(1, 177) = 200.31, *p* < .001), there were no significant differences between high (*M* = 2.28, SD = 1.17) and low (*M* = 2.37, SD = 1.13) present‐day political risk conditions for confrontational collective action intention, *U* = 3890, *p* = .49, *r*_rb_ = .06. Moreover, there were no significant differences between high (*M* = 3.39, SD = .94) and low (*M* = 3.37, SD = .97) present‐day political risk conditions for non‐confrontational collective action intention *U* = 5827, *p* = .49, *r*_rb_ = .05, whereas politicized activist identity (*F*(1, 177) = 115.73, *p* < .001) and participative efficacy were associated with stronger non‐confrontational collective action (*F*(1, 177) = 4.93, *p* = .028), Lastly, we could not find any significant interaction effect on collective action intentions. Altogether, the present‐day political risk of climate activism did not influence either confrontational or non‐confrontational climate collective action (but see Appendix S1 for detailed correlational analyses).

Overall, Study 3 failed to find causal nuanced links between risk perceptions and willingness to participate in confrontational and non‐confrontational collective action. Nevertheless, Study 3 replicated the correlational findings of the first two studies: Politicized activist identity remains the strongest predictor for both tactics. Also, explanatory correlational analyses (see Appendix S1) further demonstrated the distinct role of risk perceptions in different domains and temporality levels, such as future existential and present‐day political, on different forms of climate collective action, as in Study 2. The future existential risk of climate crisis predicts non‐confrontational action, while the present‐day political risk of climate activism drives the willingness to engage in confrontational tactics.

Nonetheless, we did not find causal links and the future existential risk of climate crisis manipulation failed. One explanation could be the very fact that the perceived risk of the climate crisis is inherently future‐oriented and is influenced by expectations regarding the timing, not just the nature or level of the crisis. This led us to conduct Study 4 where we manipulate the temporality of future existential risk of climate crisis (close vs. distant).

## STUDY 4

Building on the understanding that risk perceptions, particularly existential risk of the climate crisis, are inherently future‐oriented, Study 4 delves deeper into the temporal aspects of the existential risk of the climate crisis. To do so, it focuses not on the level of risks but on the anticipated timing of these risks. Given the urgent need for future‐focused responses to global challenges, this study explores how individuals' expectations regarding the timing of the climate crisis (close vs. distant future existential risk), in addition to the level of present‐day political risks of climate activism (as in Study 3), influence their willingness to engage in different forms of collective action. We conducted a pre‐registered 2 × 2 between‐subject factorial experiment (https://osf.io/wz3a8/?view_only=c618f7430b9d43cc98cbc7458c49cb00) as in Study 3, manipulating the present‐day political risk of climate activism (high vs. low) and temporality of future existential risk of climate crisis (close vs. distant).

### Method

We collected data online in June 2023 in Germany. We used the same data collection strategy, power analysis, and inclusion/exclusion criteria in Study 3. The final sample was *N* = 222. Participants' average age was 25.7 (SD = 11.6). Forty‐nine participants were self‐identified males, 160 were female, 10 non‐binary, and three preferred not to answer. Most of the participants were from Thuringia (*n* = 144). From 1 = left to 10 = right, participants' political orientation on average was 2.7 (SD = 1.69).

#### Measures

We measured activist identity (*α* = .87), group efficacy (*α* = .69), participative efficacy (*α* = .82), confrontational (*α* = .89), and non‐confrontational climate collective action (*α* = .83) using the same items in previous studies. Confrontational and non‐confrontational climate collective action items were from two confirmatory components as in studies 2 and 3. We also measured the present‐day political risk of climate activism (*r* = .66) as in previous studies to test the functionality of manipulation. We added one more item to the future existential risk of climate crisis measure (“How do you evaluate the likelihood of the global climate crisis destroying the vast majority of vital resources in the world”) and measured it with three items (*α* = .65).

#### Procedure and manipulation task

We used the same randomization order, procedure, and manipulation task for the present‐day political risk of climate activism as in Study 3, while we revised the failed manipulation for the future existential risk of Study 3 and focused on temporality (close vs. distant) of future existential risk of climate crises instead of its level. In temporally close vs. distant existential risk conditions, after a very brief introduction to the climate crisis, a piece from an interview with a fictitious esteemed researcher summarizes their new findings on the timing of the destructive effects of the climate crisis. In the close existential risk of climate crisis condition, the piece describes how the destructive consequences of the climate crisis are going to happen soon:

There are many signs that show that the destructive consequences of the climate crisis will hit the world in the next few years. The melting of glaciers in the European Alps appears to be progressing faster than previously thought. The number of extreme weather events has more than tripled in the past 50 years. Forest fires lasting for days and floods have already destroyed the habitats of thousands of people in Europe. We don't have time.

In contrast, the distant existential risk condition summarizes how new research findings are more optimistic than previous predictions; hence, the effects of the climate crisis will not be in the near future:

There is much to suggest that the consequences of the climate crisis will not occur so early, as we expected previously. The melting of the glaciers in the European Alps appears to be happening more slowly than we previously thought. The number of extreme weather events in Germany has increased indeed, but only slightly and slowly. The extreme forest fires and floods have not yet reached Europe. We still have time to act.

### Results and discussion

The results of the Mann–Whitney *U*‐test analysis show that participants in the high present‐day political risk condition (*M* = 2.68, SD = .90) evaluated the political risk higher than participants in the low political risk condition (*M* = 2.36, SD = 0.90), *U* = 4860, *p* < 0.001, indicating that, like in Study 3, the manipulation worked. However, we could not find the targeted difference in the future existential risk perceptions of participants who were assigned to close vs. distant existential risk conditions, *U* = 5509, *p* = .18, suggesting future existential risk manipulation failed once again.

As in study 3, we reported our analysis only for present‐day political risk manipulation since the future existential risk manipulation failed. While politicized activist identity was associated with stronger confrontational collective action (*F*(1, 215) = 178.66, *p* < .001), there were no significant differences between high (*M* = 2.41, SD = 1.08) and low (*M* = 2.52, SD = 1.15) present‐day political risk conditions for confrontational collective action intention, *U* = 5885, *p* = .567, *r*_rb_ = .04. Similarly, there were no significant differences between high (*M* = 3.55, SD = .89) and low (*M* = 3.48, SD = .95) present‐day political risk conditions for non‐confrontational collective action intention *U* = 5827, *p* = .487, *r*_rb_ = .05, whereas politicized activist identity (*F*(1, 177) = 115.73, *p* < .001) was associated with stronger non‐confrontational collective action (*F*(1, 215) = 114.97, *p* < .001), Once again, we could not find a causal link between risk perception and climate collective action intention (see Appendix S1 for correlational analyses).

## GENERAL DISCUSSION

A comprehensive understanding of climate activism is vital to assess how these movements influence decision‐makers, governments, and organizations, thereby shaping climate‐related policies and actions at local, national, and international levels. Climate activism goes beyond policies; it involves transforming societal attitudes and norms (Fisher & Nasrin, 2021; Simpson et al., 2022), including how individuals perceive their responsibilities towards the environment and future generations. Examining climate activism enables us to better grasp the broader social and cultural shifts surrounding climate change. Hence, in the context of the climate crisis, we examined the impact of (i) anticipated future risks such as the existential risk of climate activism (i.e., climate risk or *risk of inaction*), (ii) present‐day risks such as political risks (i.e., protest risk or *risk of action*), and (iii) group consciousness (i.e., politicized social identity, efficacy beliefs) on climate collective action intention in Germany across four studies.

Findings from our research consistently demonstrated that politicized climate activist identity was the most robust predictor of both confrontational and non‐confrontational climate collective action. This emphasizes the significant role that social identity plays in motivating individuals to actively engage in addressing climate change, regardless of the specific tactics and risks involved. Furthermore, our studies revealed the complex relationships between future‐oriented existential climate risks and present‐day political risks with different forms of climate collective action. While the present‐day political risk of activism was associated with a stronger willingness to engage in confrontational climate collective action, non‐confrontational collective action intention was positively predicted by future existential climate risk. Previous studies showed that, particularly in repressive contexts, fear of repercussions can stifle environmental mobilization, even when individuals are motivated by strong beliefs (Uysal, Vestergren, et al., 2024). However, activists may still employ climate collective action not only as a coping strategy against negative emotions stemming from climate concerns but also against increased repression and political risk, such as police brutality and sanctions, by utilizing different tactics that involve confrontation and self‐defence. Overall, this denotes how future‐oriented collective action is inherently grounded in the present, too; our presently held identities, and the perceived constraints and opportunities offered by our contemporary societies, shape the collective strategies we take (or do not) in bringing about a more sustainable future.

While politicized social identity remained a robust predictor between different action tactics, the content of perceived risk (future existential vs. present‐day political) in one's environment determined the transition between engagement in confrontational and non‐confrontational tactics. This aligns with the idea that collective action strategies are part of the action repertoire and do not exclude each other, forming a continuum of activist praxis (Álvarez et al., 2024; Uysal, Saavedra, & Drury, 2024; Zúñiga et al., 2023). Individuals who identify with the climate justice movement could participate in both confrontational and non‐confrontational collective action, while those who perceive high political risks, such as repressive sanctions and police brutality, could engage more with confrontational collective action. These confrontational tactics could be seen as self‐defence or more pragmatic and effective in challenging existing power structures under the pressure of political risks of activism. Meanwhile, non‐confrontational tactics, which might be more useful to gain third‐party support and succeed in attitude change among the general audience, become plausible under the pressure of the high existential risk of the climate crisis.

Nonetheless, we did not find causal links between risk perceptions and collective action. First, in both experiments, our attempts to manipulate the level and temporality of future existential risk were unsuccessful. One possible explanation is that Germany is one of the countries with the highest levels of climate concern (Uysal, Vestergren, et al., 2024), making it difficult to manipulate already salient high existential risk perceptions. Additionally, a fictional newspaper article may not be sufficient to manipulate such a deeply ingrained perception. Future studies could consider using vignettes or interactive methods to manipulate perceptions of existential risk concerning the climate crisis. Second, although our manipulation of present‐day climate activism was effective, it did not influence collective action intention. One significant reason for this could be that our conceptualization and manipulation of the political risks of climate activism were limited to police violence and legal sanctions. However, the risks associated with climate activism also encompass do‐gooder derogation (Sparkman & Attari, 2020), social exclusion, and broader activist burnout (Gorski, 2019; Gorski & Chen, 2015; Prosser et al., 2024). Therefore, a more comprehensive conceptualization and manipulation of political risks related to activism, including legal, social, political, and health‐related aspects, is essential for investigating the causal link between political risk perception and collective action.

### Limitations and future directions

While our study offers valuable insights into the relationship between risk perceptions and climate collective action, several limitations should be acknowledged. Firstly, as we aimed to reach a sample rich in terms of activists or politically engaged participants, our sample predominantly consisted of young individuals, primarily university students, which may limit the generalizability of our findings to broader populations. It is important to acknowledge that our samples consist primarily of students and activists who are generally more engaged in the climate movement than the general public. Future studies could explore the impact of risk perceptions on collective action by separately looking at the engagement of activists and the third‐party support of the general public (see Saavedra & Drury, 2019a, 2019b). Secondly, our reliance on quantitative methods limited the depth of our analysis regarding activists' perspectives and agency (see Uysal, Saavedra, & Drury, 2024 for broader discussion). Incorporating qualitative studies alongside quantitative analyses could offer richer insights into the complexities of risk perceptions and their influence on climate collective action (e.g., Haugestad et al., 2021; Vestergren et al., 2018). Moreover, our study focused solely on data collected from Germany, which may restrict the applicability of our findings to other cultural and socio‐political contexts, as context can boost or undermine the impact of psychological correlates of climate activism (Uysal, Vestergren, et al., 2024). The present‐day political risks could vary dramatically across countries. For instance, in authoritarian countries where political risks reach excessive levels, the buffering effect of identity and the emergence of confrontational collective action as an alternative for non‐confrontational ones could be weakened and even diminished. Future research could adopt a multi‐country approach to continue to explore how contextual factors influence risk perceptions and climate activism strategies across different regions.

## CONCLUSION

Although inherently geared towards the future, our correlational and experimental studies reveal that, at least in Germany, climate collective action is crucially influenced by present‐day risks and climate commitments. Specifically, our studies show that different collective action tactics are predicted by different types of risk perceptions. Although non‐confrontational action is predicted by future existential risks (risk of *inaction*), confrontational actions are predicted by present‐day political risks and sanctions (risk of *action*). Across the different studies, our research also shows that politicized climate activist identity was the strongest predictor of *both* confrontational and non‐confrontational climate collective action, illustrating the mobilizing power of group‐level psychological commitments to re‐addressing the climate crisis. Overall, our research showcases the importance of further attending to current political risks, alongside long‐term existential threats, to foster a comprehensive understanding of climate (in)action.

These findings indicate collective action tactics are not stable—and opposite—elements of certain groups, rather they should be seen as varied and rooted in social and historical contexts that predict how people perceive future‐oriented existential and present‐day political risks. Confrontational collective action is often fed by anticipation of present‐day political risk that stems from historically conflictual and/or oppressive relationships with police and crowds. Although our studies showed that there are no inherently radical crowds since different tactics are varied and dynamic options for activists, certain crowds and groups continue to be seen and oppressed as inherently radical; therefore, they developed tactics that they view as potentially more effective in such an oppressive set of relationship with law enforcement. Thus, the stable understanding of collective action tactics creates a self‐fulfilling prophecy and reinforces the conflicts (Drury & Reicher, 2000, 2009; Uysal, Saavedra, & Drury, 2024). Therefore, reaching an understanding that views collective action tactics as the dynamic and varied features of the historical and social contexts, rather than the inherent stable features of the groups, is imperative.

## AUTHOR CONTRIBUTIONS

**Mete Sefa Uysal:** Conceptualization; methodology; formal analysis; project administration; writing – original draft; writing – review and editing; data curation; investigation. **Nuria Martinez:** Writing – review and editing; conceptualization. **Sara Vestergren:** Conceptualization; supervision; writing – review and editing.

## CONFLICT OF INTEREST STATEMENT

The authors have declared that no competing interests exist.

## Supporting information


Appendix S1

## Data Availability

The data that support the findings of this study are openly available in OSF at https://osf.io/hv8ks/.
