# Supplementary material for: The horror of today and the terror of tomorrow: The role of future existential risks and present‐day political risks in climate activism
Source: Br J Soc Psychol. 2024 Nov 4;64(1):e12821. doi: 10.1111/bjso.12821 (PMC11590411; doi:10.1111/bjso.12821)
Supplement: Supplementary file 1 — Appendix S1 [file BJSO-64-0-s001.docx]

**Supplementary File**

**Results of Study 3 for Failed Manipulations**

A Mann-Whitney U test shows that participants who are assigned to future existential risk of climate crisis conditions show greater willingness to participate in non-confrontational climate collective action (*M* = 3.52, *SD* = .87) than those in low future existential risk condition, *M* = 3.24, *SD* = 1.00), *U* = 3333, *p* = .02, *r_rb_* = .19. In other words, future existential risk of climate crisis increases non-confrontational collective action intention. However, for confrontational collective action, there was no significant difference between high (*M* = 2.39, *SD* = 2.25) and low future existential risk of climate crisis conditions (*M* = 2.27, *SD* = 2.00), *U* = 3885, *p* = .48, *r_rb_* = .06.

**Correlational Analysis of Study 3**

In the correlational analysis, we performed bivariate correlation analysis and linear regression, using the mean scores of manipulation check items as they were future existential risk of climate crisis and present-day political risk of climate risk measures in studies 1 and 2. Furthermore, we added politicized climate activist identity and efficacy beliefs into the analysis. The descriptive statistics and zero-order correlations between variables are depicted in Table S.1.

| **Table S.1.** Means, standard deviations, and correlations of all measures, Study 3 | | | | | | | | |
| --- | --- | --- | --- | --- | --- | --- | --- | --- |
| Variables | M (SD) | 1 | 2 | 3 | 4 | 5 | 6 | 7 |
| 1. Confrontational collective action | 2.33 (1.07) | - | .68*** | .76*** | .29*** | .30*** | .40*** | .64*** |
| 1. Non-confrontational collective action | 3.28 (1.00) |  | - | .71*** | .46*** | .46*** | .53*** | .49*** |
| 1. Activist identity | 2.55 (.95) |  |  | - | .40*** | .39*** | .42*** | .51*** |
| 1. Group efficacy | 3.58 (.79) |  |  |  | - | .63*** | .43*** | .32*** |
| 1. Participative efficacy | 3.08 (.96) |  |  |  |  | - | .32*** | .32*** |
| 1. Future existential risk of climate crisis | 4.29 (.80) |  |  |  |  |  | - | .34*** |
| 1. Present-day political risk of climate activism | 2.55 (.96) |  |  |  |  |  |  | - |
| *Note*. *** *p* < .001 | | | | | | | | |

To test to what extent politicized climate activist identity, efficacy beliefs, and risk perceptions predict confrontational and non-confrontational collective action, we performed two linear regression analyses. As in Study 2, stronger climate activist identity (*b* = .70, *SE* = .06, *p* < .001) and present-day political risk of climate activism (*b* = .40, *SE* = .06, *p* < .001) predicted confrontational climate collective action, while willingness to participate in non-confrontational climate collective action is predicted by stronger climate activist identity (*b* = .48, *SE* = .06, *p* < .001) and future existential risk of climate crisis (*b* = .23, *SE* = .06, *p* < .001). The only difference from Study 2 is that stronger participative efficacy predicted non-confrontational collective action (*b* = .12, *SE* = .06, *p* = .035).

| **Table S.2.** Model summary of regression analyses, Study 3 | | | | | | | | | | |
| --- | --- | --- | --- | --- | --- | --- | --- | --- | --- | --- |
|  | Confrontational | | | | | Non-Confrontational | | | | |
|  | *b* | *SE* | *β* | *t* | *p* | *b* | *SE* | *β* | *t* | *p* |
| Activist Identity | .70 | .06 | .59 | 10.96 | < .001 | .48 | .06 | .49 | 8.15 | < .001 |
| Group Efficacy | -.10 | .08 | -.08 | -1.27 | .206 | .06 | .07 | .06 | .88 | .382 |
| Participative Efficacy | -.03 | .06 | -.03 | -.49 | .627 | .12 | .06 | .13 | 2.12 | .035 |
| Climate Risk | .10 | .06 | .08 | 1.53 | .342 | .23 | .06 | .22 | 3.92 | < .001 |
| Protest Risk | .40 | .06 | .34 | 6.66 | < .001 | .10 | .05 | .10 | 1.77 | .078 |
| *F* | 72.1 | | | | | 52.3 | | | | |
| *R*^2^ | .67 | | | | | .60 | | | | |

**Correlational Analyses for Study 4**

As we could not find a causal link between risk perception and climate collective action intentions, we proceeded with bivariate correlations (Table S.3) and linear regression analyses (Table S.4) as in Study 3.

| **Table S.3.** Means, standard deviations, and correlations of all measures, Study 4 | | | | | | | | |
| --- | --- | --- | --- | --- | --- | --- | --- | --- |
| Variables | M (SD) | 1 | 2 | 3 | 4 | 5 | 6 | 7 |
| 1. Confrontational collective action | 2.33 (1.07) | - | .63*** | .66*** | -.05 | -.07 | .34*** | .40*** |
| 1. Non-confrontational collective action | 3.28 (1.00) |  | - | .60*** | .12 | .03 | .33*** | .19** |
| 1. Activist identity | 2.55 (.95) |  |  | - | .09 | .06 | .33*** | .22*** |
| 1. Group efficacy | 3.58 (.79) |  |  |  | - | .46*** | -.04 | -.11 |
| 1. Participative efficacy | 3.08 (.96) |  |  |  |  | - | .12 | -.19** |
| 1. Future existential risk of climate crisis | 4.29 (.80) |  |  |  |  |  | - | .24*** |
| 1. Present-day political risk of climate activism | 2.55 (.96) |  |  |  |  |  |  | - |
| *Note*. *** *p* < .001, ** *p* < .01 | | | | | | | | |

As in previous studies, stronger climate activist identity predicted both confrontational (*b* = .78, *SE* = .07, *p* < .001) and non-confrontational climate collective action (*b* = .59, *SE* = .06, *p* < .001). While confrontational climate collective action was predicted by higher present-day political risk of climate activism (*b* = .28, *SE* = .06, *p* < .001), non-confrontational climate collective action was predicted by stronger future existential risk of climate crisis (*b* = .20, *SE* = .08, *p* = .009). Overall, we again demonstrated the association of future existential risk with non-confrontational actions, present-day political risk with confrontational actions, and politicized activist identification with both forms of action.

| **Table S.4.** Model summary of regression analyses, Study 3 | | | | | | | | | | |
| --- | --- | --- | --- | --- | --- | --- | --- | --- | --- | --- |
|  | Confrontational | | | | | Non-Confrontational | | | | |
|  | *b* | *SE* | *β* | *t* | *p* | *b* | *SE* | *β* | *t* | *p* |
| Activist Identity | .78 | .07 | .58 | 11.37 | < .001 | .59 | .06 | .53 | 9.18 | < .001 |
| Group Efficacy | -.08 | .09 | -.05 | -.88 | .379 | .15 | .09 | .11 | 1.73 | .085 |
| Participative Efficacy | -.06 | .07 | -.05 | -.83 | .406 | -.07 | .07 | -.06 | -.99 | .324 |
| Future existential risk of climate crisis | .16 | .08 | .10 | 1.93 | .056 | .20 | .08 | .16 | 2.66 | .009 |
| Present-day political risk of climate activism | .28 | .06 | .23 | 4.46 | < .001 | .03 | .06 | .03 | .53 | .599 |
| *F* | 45.7 | | | | | 26.8 | | | | |
| *R*^2^ | .51 | | | | | .38 | | | | |
